# Recognition of Abnormal Chest Compression Depth Using One-Dimensional Convolutional Neural Networks

**DOI:** 10.3390/s21030846

**Published:** 2021-01-27

**Authors:** Liang Zhao, Yu Bao, Yu Zhang, Ruidong Ye, Aijuan Zhang

**Affiliations:** 1 School of Mine, China University of Mining and Technology, Xuzhou 221116, China; zhaol@cumt.edu.cn; 2 Department of Computer Science and Technology, China University of Mining and Technology, Xuzhou 221116, China; zhang255086@gmail.com (Y.Z.); yerundong2020@126.com (R.Y.); zaj@cumt.edu.cn (A.Z.); 3 Mine Digitization Engineering Research Center of Ministry of Education of China, Xuzhou 401121, China

**Keywords:** convolutional neural network (CNN), chest compression classification, accelerometer sensor application, cardiopulmonary resuscitation

## Abstract

When the displacement of an object is evaluated using sensor data, its movement back to the starting point can be used to correct the measurement error of the sensor. In medicine, the movements of chest compressions also involve a reciprocating movement back to the starting point. The traditional method of evaluating the effects of chest compression depth (CCD) is to use an acceleration sensor or gyroscope to obtain chest compression movement data; from these data, the displacement value can be calculated and the CCD effect evaluated. However, this evaluation procedure suffers from sensor errors and environmental interference, limiting its applicability. Our objective is to reduce the auxiliary computing devices employed for CCD effectiveness evaluation and improve the accuracy of the evaluation results. To this end, we propose a one-dimensional convolutional neural network (1D-CNN) classification method. First, we use the chest compression evaluation criterion to classify the pre-collected sensor signal data, from which the proposed 1D-CNN model learns classification features. After training, the model is used to classify and evaluate sensor signal data instead of distance measurements; this effectively avoids the influence of pressure occlusion and electromagnetic waves. We collect and label 937 valid CCD results from an emergency care simulator. In addition, the proposed 1D-CNN structure is experimentally evaluated and compared against other CNN models and support vector machines. The results show that after sufficient training, the proposed 1D-CNN model can recognize the CCD results with an accuracy rate of more than 95%. The execution time suggests that the model balances accuracy and hardware requirements and can be embedded in portable devices.

## 1. Introduction

Chest compression (CC) is a necessary form of first aid measure, and the depth of CC is subject to precise requirement. As shown in the most recent cardiopulmonary resuscitation (CPR) manual, adequate CC depth (CCD) corresponds to a higher survival rate. The recommended CCD is approximately 5 cm, which avoids excessive (exceeding 6 cm) or insufficient (below 5 cm) CCDs during CPR [1]. However, it is difficult to maintain a high-quality CCD owing to the potential tension, fatigue, and injury risks of patients during CPR. To improve the quality of CPR training, a portable training device is used to provide feedback for correcting non-standard CC movements.

At present, various forms of auxiliary CPR equipment have been developed for measuring CCD [2]. The key technologies of these devices were designed to monitor the process of CCs, and numerous methods have been proposed to support this. One crucial challenge is assessing the accuracy of the moved CCD. Solving this can help rescuers or trainees to know whether their CCs are valid. Methods for accurately measuring depth include ultrasonic/impulse radio-ultra wideband (IR-UWB) ranging, laser ranging, infrared ranging, and accelerator sensors. In ranging sensor devices, the displacement sensor itself is bulky and difficult to install, and it does not meet the requirements of component miniaturization, portability, and power efficiency. Similar problems are in the IR-UWB and laser devices. Moreover, the IR-UWB and laser device functions use wireless signals to transmit to the CC distance and measure the time it takes to complete the radar process. The time difference of arrival can produce higher accuracies; however, its algorithm is inferior to the quadratic integration method [3]. Furthermore, clothes can sometimes render the IR-UWB results inaccurate [4]. Regarding the pressure sensor-based auxiliary CPR equipment, it has been highlighted that the measurement becomes biased if CPR is conducted on a mattress [5].

Regarding the accelerometer sensor, several studies have shown that the quadratic integration method for micrometers using a CC depth-measuring acceleration sensor is effective [6,7]. Traditional accelerometer sensor-based CCD calculation methods often use quadratic integration. This method is prone to error accumulation and numerical drift, and the final calculation results have unpredictable deviations. Furthermore, when CPR is performed on a bed, the obtained CCD result may exceed the actual one, owing to the additional acceleration effect of the mattress movement [8]. A sloping floor or bed also affects the accelerometer sensor results [9]. In such cases, an error compensation method is often used to correct the deviation. This makes most precision CPR training equipment bulky and expensive (see, e.g., in [2]), because the compensation methods involve complex calculations and therefore require extra computational resources. In [10], a Kalman filter was implemented by an external computer, to reduce and eliminate the effects of noise in the CCD results. The author of [11] recommended adding a flexible real-time pressure sensor to facilitate CC recognition, and they also connected the device to a computer to calculate the result. Therefore, our objective is to reduce the complexity of CCD calculations without reducing the accuracy of CCD evaluations.

Because accelerometer sensors are cheap and lightweight, they help to reduce the volume of CCD measurement equipment. Accelerometer sensor data can record acceleration signals to reflect hand movements in CPR. These are similar to the physiological signals monitored by medical equipment, which can also be recognized using artificial intelligence (AI) classification algorithms. Recent studies into deep learning with physiological one-dimensional signal data, (e.g. electromyogram (EMG), electrocardiogram (ECG), electroencephalogram (EEG), and electrooculogram (EOG) data) have demonstrated its high potential [12]. In [13], a long short-term memory (LSTM)-based autoencoder (AE) network was integrated with a support vector machine (SVM) for ECG arrhythmia classification; there, the LSTM-based AE network extracted ECG signal features, and the SVM classifier was applied for classifying different ECG arrhythmia signals. The results show that the proposed method achieves more than 99% accuracy. The author of [14] proposed methods for detecting pathological voices from among healthy speech data, using glottal source information. Here, two combination of methods were used: a combination of a convolutional neural network (CNN) and multilayer perceptron, and a combination of a CNN and LSTM networks. Their results were superior to those obtained by the best traditional pipeline systems. Other studies have drawn the same conclusions [15]. In [16], a single high-density surface electromyography (HD-sEMG) dry electrode device was constructed using a matrix of sensor nodes. A triple-layer CNN with a majority vote on five successive inferences was used to recognize eight hand postures, achieving an accuracy of 98.15%. Using a similar method, Maachi et al. [17] placed 18 sensors on patients’ feet, and each signal was input into the one-dimensional ConveNet of deep neural networks (DNNs). Important clinical spatio-temporal gait features (e.g., swing phase, stance phase, and stride time) can be derived from vertical ground reaction force signals to distinguish the symptoms of Parkinson’s disease. These studies implementing AI show that it is more effective to use classification methods to judge medical monitoring signals than calculation procedures.

By analyzing previous studies, we found that by changing the decision-making process, we can focus more on the types of CPR results and their classification, that is, we can consider evaluation results as good or bad, instead of using the judgment method of the compression distance calculation. CC is commonly understood to be a typical regular reciprocating motion in which the sample value of each cycle return to zero. This suggests a practical opportunity for developing monitoring equipment in the field of emergency cardiopulmonary resuscitation. Using accurate measurement of the length and amplitude characteristics of the waveform formed by the acceleration measurement values, a classification method can distinguish excessive or insufficient CCDs. Thus, we can choose a sampled data classification method to solve this problem. This is the motivation of our research.

In this study, a micro-low-power acceleration sensor is used to trace the movements of the CC. Because CPR training is conducted on emergency care simulator (ECS) that can effectively trains rescuers and prevents injuries during training, the training data we collected were from an actual ECS. Our main contributions is to change the method of CCD measurement calculation into a waveform evaluation-based operation. We adapted the max-value alignment of the training data according to the features of the CC samples. Finally, we compared the errors between our method and accurate traditional measurements, and we designed a computationally inexpensive one-dimensional CNN (1D-CNN) model that can run on CPR portable devices and does not reduce the accuracy of the CCD evaluation.

The remainder of this paper is organized as follows. In Section 2, we introduce the methods used to collect and experiment upon the training data, preprocess the data to filter the noise, establish a 1D-CNN model based on LeNet5, and improve its performance and precision. In Section 3, we experimentally evaluate the effects of different filter size, and we discuss and compare the different structures of convolutional networks. We present a discussion of our method in Section 4 and our conclusions and suggestion for future work in Section 5.

## 2. Methods

### 2.1. Data Preparation Procedures

**Figure 1:**
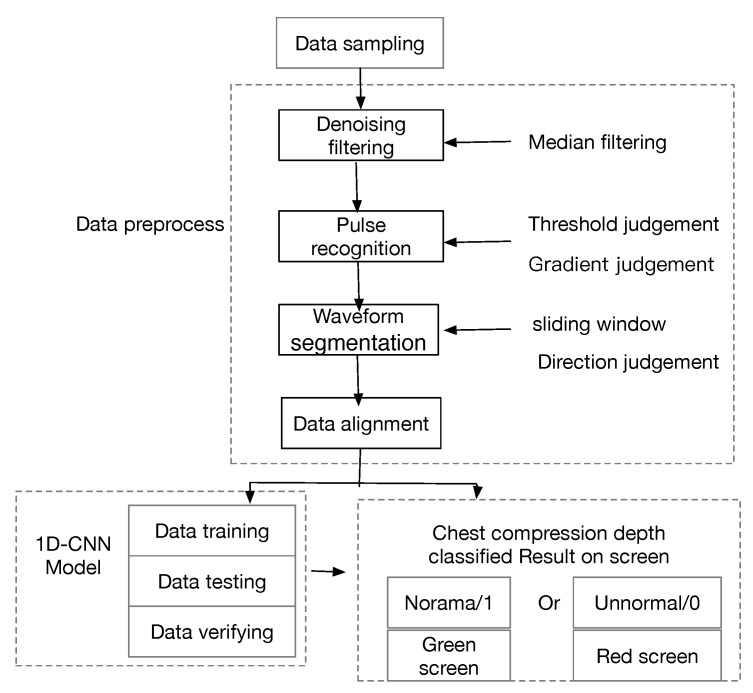
Data processing to identify normalized waveform data.

Before establishing the model, several preliminary procedures must be completed, including data sampling and data preprocessing. These are shown in Figure 1.

#### 2.1.1. Training Data Collection

**Figure 2:**
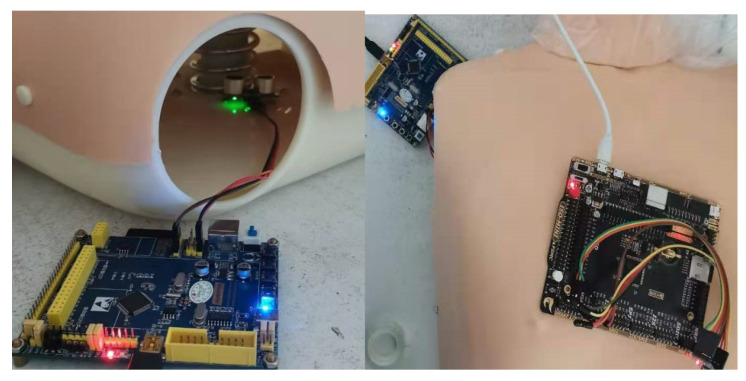
Training data acquisition setting.

To obtain the normal and abnormal CC signal data for the experiment, a CC simulation system was designed to collect acceleration data during CC process. The entire compression data acquisition system is shown in Figure 1; it can be divided into four parts: the simulation device, control module, measurement module, and sensor module. The simulation device can simulate the different pressure waveforms generated by the compression simulation process. The control module conducts data analysis, processing, and archiving. All compression data are recorded on a digital memory (SD card). The measurement module obtains the computed distance values to compare them against the results acquired using the classification methods. The sensor module conducts compression information monitoring and measurement when compression is performed to collect acceleration data from the CC process. In the sampling process, a stm32 and an ADXL345 hardware, and a v-wo smart watch with a bm250 three-axis accelerometer are used as two combinations. ADXL345 is a small, ultra-thin, ultra-low power consumption, 3-axis accelerometer with a maximum resolution of (13-bit) measurement of ±16 g. The accelerometer bm250 is similar to ADXL345, but uses a 10-bit binary value. We use the ADXL345 to obtain simulation data and then transplant the trained model to the smart watch for verification. The acquisition setting are shown in Figure 2.

#### 2.1.2. Data Preprocessing

After sample collection, the data are preprocessed to filter out noise. This study simulates an actual CC scenario during the data collection process. Each dataset contains the acceleration curves collected during continuous compression. However, the time-series data are affected by electromagnetic and voltage interference, and the raw data contain large quantities of white noise. Therefore, data preprocessing is required, including noise reduction filtering, pulse recognition, waveform segmentation, and fitting, as shown in Figure 1.

To filter white noise contained in the collected data and reduce many burrs presenting in the raw data, several filtering methods have been tested. The effect of the single-dimensional Kalman filter was notably superior. However, its operation time and complexity were high, which produced an operational delay in the microcontroller. To consider the case of small losses, 1D median filtering and low-pass amplitude-controlled filtering are adopted in this study. As shown in [18], this is consistent with the results of other previous studies.

**Figure 3:**
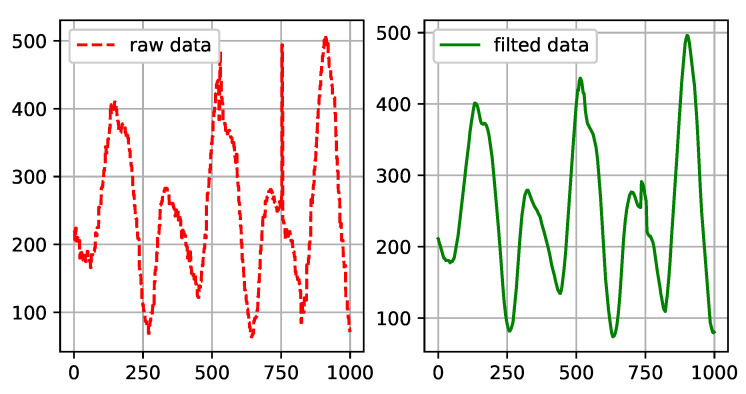
Cardiopulmonary resuscitation (CPR) data filtering. The right-hand image is obtained after filtering the left-hand data.

To analyze the raw data, low-pass amplitude-controlled filtering was first used to filter out unsuitable white noise and hammering anomalies prior to waveform analysis. After passing the data through the low-pass filter, a significant correlation could be seen between adjacent sampling points; the narrower the filtering bandwidth, the stronger the correlation. Then, we applied median filtering via a sliding window. This suppresses periodic interference and offers high smoothness. The waveform generated from the CPR data before and after filtering are shown in Figure 3.

#### 2.1.3. CC Pulse Recognition

Here, gradient and amplitude feature calculation methods were used to identify the pulse; then, the sliding window was used to distinguish the waveforms of each pressing pulse. The waveform’s width and height constrain the time consumption and strength of the CC, respectively. The number of compressions per minute determines the wavelength of a pulse. At a sampling frequency of 5 ms, the number of CC per minute is between 100 and 120. Considering the vibration of returning to the highest point and identification calculation time, the effective sampling point number is set as 70 to ensure that the downward process of each CC is complete. Then, we divided the extracted acceleration data of the waveform into the normalized input of the 1D-CNN model. The specific process is as follows.

Step 1: Establish two sliding windows. One (Sliding window A) stores the sampled value, and the other (Sliding Window B) stores the filtered result.
Step 2: Conduct threshold monitoring on the Sliding Window B and determine the threshold value when the static gravity value shows a change of α% (according to the statistical results, α experience value is between 21 and 32).
Step 3: Cut the wave in Sliding Window B. Select the optimal point from Window B or A as the starting point and the pulse end point as the termination point; physically this describes a compression process. In the cutting wave, the hand speed is zero when the compression reaches the lowest point position.
Step 4: Identify the CC pulse according to three restrictions.

Thus, a set of standardized waveforms are obtained. Using this method, pulse identification and waveform segmentation were sequentially performed on 18 sets of experimental data; finally, 937 CC waveforms were obtained.

### 2.2. Solution Based on 1D-CNN Model

#### 2.2.1. One-Dimensional LeNet5 Model

To classify CC results, 1D-CNN, a deep learning method, was used because its computational efficiency is superior to that of a recurrent neural network (RNN). Two dimension CNNs (2D-CNNs) are widely applied to tackle numerous image and video recognition problems in machine vision. The 1D-CNN performs convolutional calculation on a 1D signal [19]. 1D-CNN is a good model because 1D filters can detect different spatial shapes in one dimensional matrix [20]. 1D-CNN utilizes several 1D convolutional layers followed by max-pooling layers, and dynamic fully connected layers with ReLu activation functions. Dropout filters are utilized after the first FC layers with a probable value. The input of our CNN model is an array representing the CPR waveform, which is denoted as *X*. The network is designed to learn a set of parameters to map the input to the prediction *Y* according to a hierarchical feature, which is given by Equation (1):

(1) $$Y=F\left(X|\Theta \right)=h_{L}\left(W_{L}\ldots h_{1}\left(WX_{n}+b_{1}\right)+b_{L}\right),\Theta_{L}=\left[W_{L},b_{L}\right].$$

where *L* is the number of hidden layers in the network and $l_{N}$ represents the length of the convolution kernel of layer *l*. The array contains one dimensional data; thus, $X_{n}$ is a 1D input matrix of N feature maps, *W* is a set of N 1D kernels used to extract a set of features from the input values, *b* is a bias vector, and *h*() is an activation function. The convolution layer calculation formula is defined as

(2) $$y_{i,j}^{\left(l\right)}=\sum_{i=1}^{1}\sum_{k=1}^{l_{N}}w_{i,k}^{\left(l\right)}x_{i,j+k}^{\left(l-1\right)}h^{\left(l\right)}+b_{i}^{\left(l\right)}.$$

For simplicity, we define two classes of CCD results: 0 is an error (excessive or insufficient distance) and 1 denotes a correct result. Thus, the problem becomes one of binary classification. Then, the cross-entropy is defined in Equation (3):

(3) $$L_{H}=-\frac{1}{N}\sum_{k=1}^{N}\left[{\hat{y}}_{k}logy_{k}-\left(1-{\hat{y}}_{k}\right)log\left(1-y_{k}\right)\right]$$

where $\hat{y}$ is the label value of the sample.

**Figure 4:**
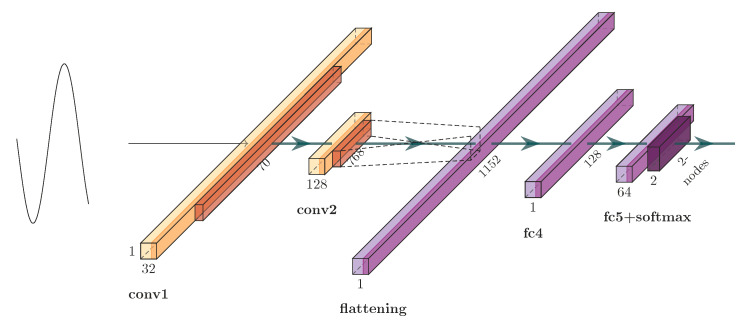
1D-LeNet-5 model.

The number of CNN parameters directly corresponds to the computational cost of training and the demand for large amount of training data. In particular, the recognition component of CCD classification is used for devices with low computational capabilities. Therefore, we attempt to determine a simple neural network structure for training. 1D signals are typically used in monitoring tasks, and Pytorch contains a special function for them. We chose a 2D-CNN to modify for this case, because we hope to extend the sensor type in the future. We first considered the LeNet-5 [21] model, which was originally used to recognize handwriting inexpensively and with a simpler network structures. The 2D-CNN LeNet-5 architecture consists of two sets of convolutional and average pooling layers, followed by a flattening convolutional layer, two fully connected (FC) layers, and a softmax classifier [21]. The convolution layer is the local perception feature of the CPR signal, that is, it perceives features of each part of the signal. Next, a more comprehensive operation is performed to obtain global information. This reduces the computational parameters of the model via Equation (4):

(4) $$Z_{i,j}^{\left(l\right)}=\sum_{i=1}^{1}\sum_{k=1}^{m}x_{i,j+k}^{\left(l-1\right)}{\hat{w}}_{i,k}^{\left(l\right)}$$

where *Z*, *x*, and $\hat{w}$ represent the convolution result, waveform data value, convolution kernel coefficient, respectively. *i*, *j* and *k* are location. The second dimension is set to 1. However, LeNet-5 is designed for 2D image recognition of handwriting. We set one of the dimensions as 1, to render the model applicable to the one-dimensional case, and the adapted model is shown in Figure 4.

The architecture shown in Figure 4 is composed of two convolution layers (with a filter length of five) interlaced with two max-pooling layers (not drawn), and followed by two FC layers and an output layer. The stride was set to one step. A ReLU activation function (h(x)=max(x,0)) was used for all layers except the output one, where a softmax classifier was used to output the posterior probability of each class. Finally, the FC layer (containing 128 neurons) and output layer were used for fault detection and classification. The three convolution layers contained 32, 64, and 768 neurons, respectively.

The 1D raw CC data from accelerometer sensors were preprocessed before being input to the 1D-CNN classifier for learning. The aforementioned data preprocessing normalized the data and distinguished each positive CC as a wave signal for 1D-CNN inputting.

#### 2.2.2. Data Feature Analysis and Labeling

In the actual emergency CPR process, it is necessary to repeatedly press multiple times within a short period. Inevitably, problems such as obstruction and jitter occur during distance measurement. Moreover, in most cases, it is difficult to achieve high-precision millimeter-level measurements; therefore, it is difficult to obtain a large number of CCD data labels based on precise distances. To solve the problem of accurate training set labels, we used a high-speed camera and an ultrasonic rangefinder to make correction. Ultrasonic ranging can achieve an accuracy of 1 mm when measuring short distances. An ultrasonic rangefinder was installed inside the ECS, to measure the compression distance.

**Figure 5:**
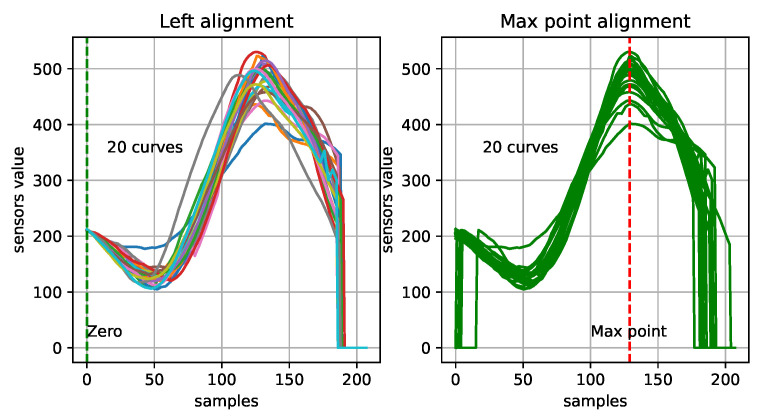
Data alignment of normalized waveform data. The waveform of the left-hand figure is aligned by start points, with that of the right aligned by maximum point.

After data preprocessing, the cutting length of the waveform generated by each CPR did not match the sampling data number, and the input data had to be normalized to the length of the 1D-CNN model’s input. We filled each input data entry with 0 or eliminated any extra data to make it fit the input dimension of the network model. The input data alignment determined the location of the data zero filling. Figure 5 shows the two filling methods that are used to adapt the model input to different wavelengths and sampling rates: (1) data aligned at the beginning and zero-filled at the end, and (2) data aligned at the position of maximum value and zero-filled at both sides. Because the former disperses the waveform gradient area and makes it difficult to learn the gradient change of the CPR signal wave, we adopted the latter. To prevent missing values, each curve was centered on the largest point, with 75% of the points on the left and 25% of them on the right, to form a dataset. After processing, the model input data has the same dimensions.

Preliminary analysis of the data curve containing the label and maximum alignment graph shows that the wave similarity is high when they denote a valid CCD. When the gap between the compression distances exceeds 5 mm, a large jitter occurs in the center of the compression signals. If the interval between two compressions is too long, the waveform display is abnormal compared to the standard one. Furthermore, several visibly correct waves that were concentrated on the two extreme points of the wave set were too smooth or too prominent. Therefore, the variance at the two extreme points was used to measure the degree of discreteness of the data, which may be another important characteristic.

#### 2.2.3. Improvement of 1D-LeNet5 Model

After training, the testing results were promising compared to our previous simulation [18]. However, the accuracy rate was reduced when we used the model in the ECS. We hope to enhance the model by improving upon the previous 1D-LeNet.

**Figure 6:**
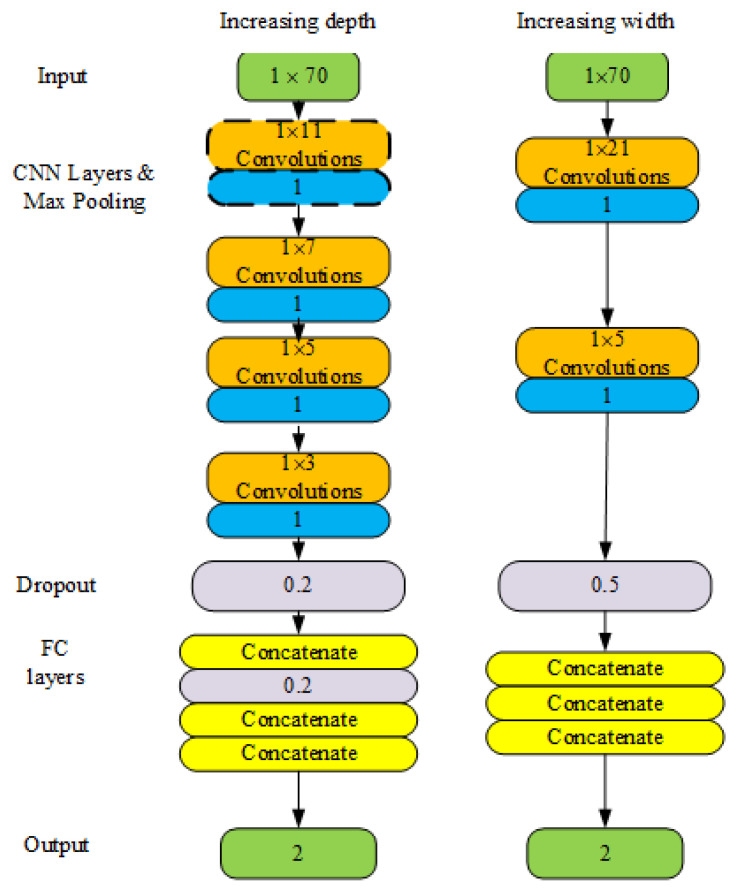
Illustration of improving 1D-CNN architecture. Each rectangle represents a layer in the neural network. For convolutional neural network (CNN) layers, filter size is shown, and the same layer is painted the same color. CPCNN4 did not has convolution layer 1×11 denoted by dotted boxes.

The basic structure of the 1D-CNNs included convolution layers, pooling layers, and FC layers. Different CNN structures have different effects on the signals. Based on these layer types, we tested two ways to improve the original model. First, according to the works in [22,23], deeper networks typically offer better performance. Considering the running environment of the CPR program, we added one or two hidden layers, which may be feasible at a low costs. If this was proved effective, we tried to add three or more hidden layers and tested whether the device with low computational capability could respond in time. Second, in CNN, the large filter size allows large receptive fields to be obtained [19]. However, several studies have shown that stacking a small number of filters may achieve the same goal and offer computational benefits. If two layers are stacked, nonlinearities should be inserted between them; this increases the representational power of the CNN and subsequently improve accuracy. We compared the two methods. Third, if there are large receptive fields in the first convolutional layers, this layer is assumed to have a more global view of the wave signal. Moreover, the electronic noise is non-stationary, that is, the frequency or spectral contents of the noise are stochastic and like a pulse. Therefore, shorter filters do not provide a general view of the spectral contents of the signal and can easily absorb the noise signal, yielding indecision. We enlarged the filter of the first layer and compared it with the others. Two improvement ideas are shown in the Figure 6. At the beginning, the filter scale multiple of increasing depth method is the same as that of increasing width to compare the performance of them. To avoid overfitting, batch normalization and pooling should be performed after the activation function of each convolution layer. Dropout is an effective technique to address the overfitting problem of deep neural nets [24], the key idea of which is to randomly drop units and their connections from the neural network to prevent co-adapting between units. We used dropout filter after the last convolution layer and first FC layer, as shown in Figure 6. Considering the small number of features, we did dropout at the two places in order to facilitate the probabilities adjustment of dropout at different layers.

## 3. Experiments and Results

We performed three experiments to compare several parameters of the 1D model. The 1D-CNN model was constructed using the Pytorch framework [25]. We acquired 937 CCD records with an error of ±1 mm made by 9 people on a tables. Among them, we selected 680 records as the training set and 120 records as the testing set; the rest were used for validation. Correct CPR records accounted for a half (471), and excessive and insufficient error records accounted for the remaining half. The batch size was 40. The first experiment compared filters of different sizes; the second compared the different numbers of network layers and identified which was better for low computation capability devices. The final experiment compared the performances of different 1D-CNN models on the dataset.

For the sampled data, the network outputs the results of the two classifications through the FC layer, which determines whether the sampled data meet the CC criteria. We used the metrics of accuracy (accuracy, Acc) and F-Score [26] to evaluate the network classification performance. Their respective definitions are given as

(5) $$ACC=\frac{\left(TP+TN\right)}{\left(TP+TN+FP+FN\right)},$$

(6) $$F-Score=2\times \frac{\frac{TP}{TP+FP}\times \frac{TP}{TP+FN}}{\frac{TP}{TP+FP}+\frac{TP}{TP+FN}}.$$

Here, *TP* denotes the true or actual normal data classified as normal data; *TN* denotes true abnormal data, which are correct data classified as abnormal; *FP* denotes the false normal data, which are abnormal data classified as normal; and *FN* denotes the false abnormal data, which are normal data classified as abnormal. In statistical analyses of binary classification problems, the *F*-*score* indicates the accuracy of the test; it represents the harmonic mean of the precision and recall. An *F*-*score*’s optimal value is 1, and its worst value is 0 [27].

### 3.1. Comparison of Different Filter Size

In this study, we designed several sets of comparison experiments, to test the relationship between feature extraction and filter size. The results of 50 experiments of each line are summarized in Table 1. Table 1 shows that the deep features extracted by these filters with different sizes exhibited positive and negative recognition rates under different learning loop times; this indicates that the feature extractor can filter out the correct CCD well and can adapt favorably to the network characteristics. However, the results also show that the 1D-LeNet5 model can easily fall into a local solution when a small filter size is used. When we enlarged the filter size, 1D-LeNet5 can escape the local solution. Furthermore, the wide filter size outperforms the small one in terms of learning time and recognition accuracy. The small size filter network sometimes falls into a local solution when the initial filter is created with a random value.

### 3.2. Comparison of Different Numbers of CNN Layer

To enhance the results, we used the proposed 1D-CNN and then added several new hidden layers to the original neural networks to form a six or seven layer CNNs. Then, in the CNN structure, the convolution and max pooling layers were added and placed them alternately through the network. The last layers were fully connected to obtain the output. We compared the network performance under different layers to find the ideal number of CNN layers for embedded systems.

**Figure 7:**
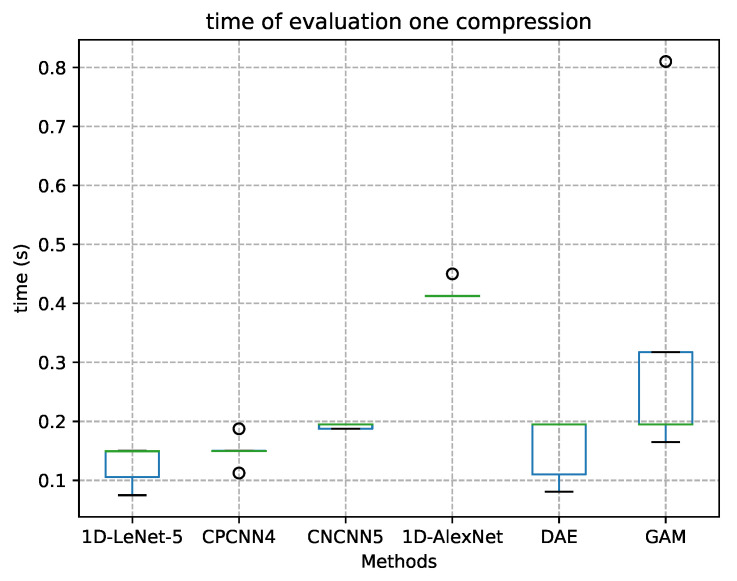
Time cost for different layers and filters.

We chose the AlexNet model because it has the closest number of layers to LeNet5 and offers better performance. This model contains eight layers: the first five are convolutional layers, and the last three are FC ones. In subsequent experiments, AlexNet achieved a high accuracy rate. The experimental results of 50 times for each filter shape method presented in Table 2 indicated that the increase in convolution layers increased the learning time and improved the recognition accuracy of the signal for the samples in the dataset. We could not add too many convolution layers because the CC recognition should be completed in a very short time (no more than 600 ms). The 1D-LeNet5’s computation time was between 110 ms and 160 ms on our 240 MHz embedded device. We compared the running times of one sample recognition in Figure 7. The time was obtained using the same CPU time. Thus, from Figure 7, we can see that 1D-AlexNet may require more time for CC recognition, which suggests that the ideal number of convolution layers is five or six.

### 3.3. Comparison of Different Methods

Here, we compare several other CNN models, including AlexNet and KNN. 1D-AlexNet was obtained by setting one of the dimensions of the 2D model [28] to 1. Table 3 shows the average classification accuracies achieved by multiple 1D-CNNs, as well as the results achieved by other state-of-the-art methods described in the literature. In the worst case scenario, involving a falling into local optimum, the average accuracy of our proposed 1D-CNN with for convolution layers was 94.79%. Our other setup, with 4 convolution layers, performed better: its average accuracy was 96.67%, and its deviation was only 0.26% in ten folds.

The proposed CC 1D-CNN with three or four convolution layers (CPCNN4 or CPCNN5, respectively), denoising autoencoder (DAE) network (DAENet) [29], and GammatoneNet [19] are all 1D-CNNs, which learn the representation directly from the signal. Heart sounds were extracted using the DAE algorithm and used as the input feature of the DAENet. The periods of the heart sound signals were very similar to the compression signals in waveform; GammatoneNet use 2D and 1D representations of the audio signal as input, achieving a good performance on 1D signals. Therefore, the two methods may be suitable for our project.

**Figure 8:**
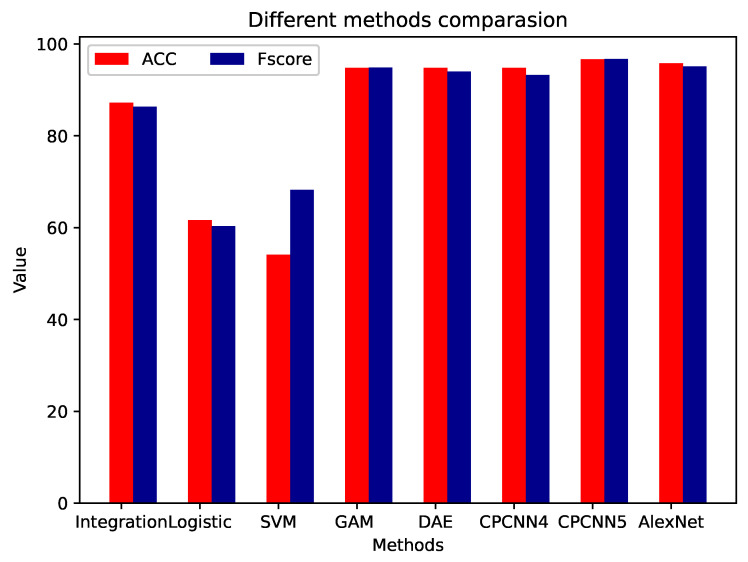
Precision comparison for different layers and filters.

**Figure 9:**
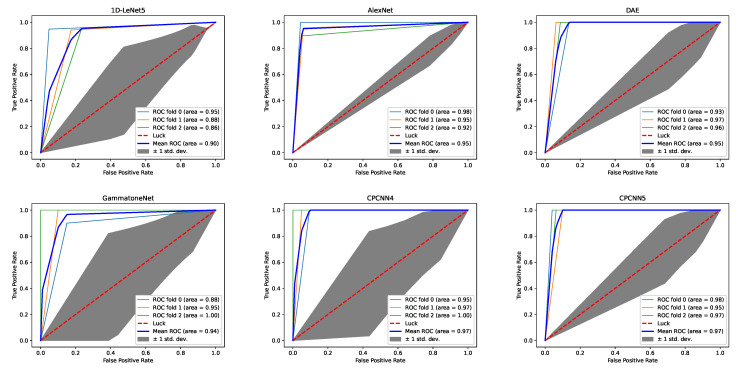
ROC curves of various 1D-CNN models.

In Table 3, we list the network structure, filter size, the number of parameters, and floating point operations (FLOPs) of all methods. The proposed method was no more structurally complicated than methods of a similar accuracy, and it used fewer parameters and more FLOPs than the GammatoneNet methods described in the literature. However, as shown in Figure 7, the CPCNN5 method does not take much more time than other methods, and it can still be used on embedded devices. In Figure 8 and Table 3, we compare the ACC and F-score for the experiments. Our method outperform the general classification and integration method for the collected samples, and it can be embedded into low devices. The receiver operating characteristic (ROC) curves were plotted for these models to assess the performance using an alternative metric: the area under the curve (AUC). The AUC value of an ideal classifier is “1”, and a random guess gives an AUC value of 0.5 (shown as a dotted line on the ROC plots in Figure 9). Our CPCNN4 and CPCNN5 model provided a higher mean AUC value of 0.97, outperforming the other models considered here.

## 4. Discussion

Because the data of waveforms need to be buffered for filtering, we first cut the waveforms of CCs and turned the time series data into the features of waveform data. Therefore, the CNN model was chosen. The experimental results show that the features extracted using the proposed model have clear discriminability; furthermore, these features are clearer in the amplitude and time series of the sampling signals, which improves the classification. The number of CCs per minute is between 100 and 120 [1], which limits the time of one compression between 500 ms and 600 ms. Considering the intervals between two compressions, the waveform length of each compression data is limited by sensor’s sampling frequency, and the size of a filter will also be limited. According to experimental experience, when a filter exceeds half of waveform length, the learning effect is poor. Although a large convolution kernel increases the computational complexity, it can easily obtain features when the number of convolution layers is small. In Table 2, the convolution kernel sizes selected are typically prime number, and the effect of the prime size filter is superior to that of the filters with a normal convolution kernel size. Comparing the results in Table 3 to those of our previous study, we can confirm that although the hardware error and performance cannot be changed, the classification method based on 1D-CNN is more accuracy than the method based on quadratic integral calculation, and it does not require additional equipment either.

In our designed deep networks, as the number of layers increases and other designs remain unchanged, the number of calculations increases slightly, and the time required for classification and identification is extended. In some cases, the computational capabilities of embedding device may be inadequate. According to Table 3 and Figure 7, the number of parameters seems to have a greater impact on the performance of these model than FLOPs, which needs to be considered when choosing a model. The network architecture consisting of CPCNN4 or CPCNN5 convolutional layers, depending on the processing capabilities of the device, may provide a suitable choice. In the learning process of our experiments, the GammatoneNet, AlexNet, and CPCNN5 models do not easily fall into the local optimum situation (i.e., they did not converge before reaching the maximum number of iterations in 50 experiments, respectively), whereas the ACC scores of LeNet5 models may converge early in multiple scenarios. The results show that the likelihood of our method falling into a local optimum is small.

Although our research performed well in identifying most CCDs, several boundary values (CCD is near 5 cm or 6 cm) were still misjudged. A possible reason is the nuances of these features have not been extracted in the model, and inception model or LSTM may be helpful to them. It needs to be tried further. Regarding the collected data set, these data were collected on ECS because we were worried about possible personal injury caused by an experimental equipment. Although these data may be different from real CPR data, this method can be applied to real scenarios after training on real CPR data. We will collect real human body CPR data to further verify the effectiveness of our work. Because it is difficult to collect real human bodies CPR data accurate to millimeters, the effective use of unsupervised learning with inception model might be another way [30]. In future work, we will compare whether the LSTM model performances better on this problem.

## 5. Conclusions

This paper proposed a 1D-CNN-based method to replace the CCD measurements and assessed its feasibility. By comparing different network performances, the method was evaluated on a data set of 937 chest compression samples. From the experimental analysis, the following conclusions can be drawn. The proposed classification method outperforms the quadratic integral measurement with a 9.4% higher ACC; it also outperforms SVM and other methods considered in this paper. The modified 1D architecture network outperforms the 1D-LeNet5 method (in terms of accuracy) and the 1D-ALexNet method (in terms of calculation time); moreover, it does not require extra computational auxiliary devices.

To summarize, the model proposed here offers an improved recognition accuracy for regular reciprocating motion measurements, and it is suitable for embedding in the portable measurement equipment used to evaluate such motions (e.g., cardiac compression orthosis). In future work, we will verify the feasibility of this combination and determine whether it can achieve a better performance in monitoring-data detection. In addition, to determine whether the model can be applied to lower-end devices, it is necessary to further investigate methods of reducing the hardware requirements of such tasks, and to further improve the performance of the 1D-CNN.

## Figures and Tables

**Table 1:** Comparison of test results for different 1D convolution filter sizes.

1st Convolu-Tional Filter Size	Second Filter Shape	Iteration	Max/Min/Avg Accuracy Rate (%)	Max/Min/Avg F-Score (%)
5	1 × 5	500	95/65.83/82.62	95.16/73.55/88.488
5	1 × 5	1000	92.5/65/87.64	92.91/73.08/88.534
5	1 × 5	1500	95.83/65.83/88.166	95.93/73.55/89.918
5	1 × 5	2000	95/91.67/93.334	95.16/91.53/93.466
7	1 × 5	500	93.33/90.83/92	93.65/90.91/92.27
21	1 × 5	500	95/93.33/94.166	95.16/93.44/94.208

**Table 2:** Performance comparison for different numbers of 1D convolution layers.

Filter Shape	Iteration	Max/Min/Avg Accuracy Rate (%)	Max/Min/Avg F-Score (%)
1×7,5	500	93.33/90.83/92	93.65/90.91/92.27
1×7,5,3	500	97.5/90/94.79	9.52/89.29/94.62
1×11,7,5,3	300	99.17/95/96.67	99.15/95.08/96.7
1D-LeNet	2000	95/91.67/93.33	95.16/93.44/94.208
1D-ALexNet	300	95/93.33/94.305	95.16/93.22/94.208

**Table 3:** Performance comparison of different numbers of 1D convolution layers.

Methods	Average ACC	Convolution Layers	Filter Shape	Total Param	Total FLOPs
Integration	87.2	-	Median filter	9	7*200
Logistic	61.66	-	Sigmoid	-	-
SVM	54.1	-	RBF	-	-
Lenet-5	93.33	32*64*1088*128*64	1×5,5	$1.58\times 10^{5}$	$1.75\times 10^{6}$
1D-AlexNet	95.8	32*64*128*256*1024	1×11,5,3,3	$1.1\times 10^{6}$	$4.17\times 10^{6}$
GammatoneNet [19]	94.82	16*16*32*64*128	1×16,8,4,2	$2.7\times 10^{5}$	$2.21\times 10^{6}$
DAE [29]	94.8	32*12*24*132	1×13,13	$3.08\times 10^{4}$	$6.4\times 10^{5}$
CPCNN4	94.79	32*64*128*1152	1×7,5,3	$1.91\times 10^{5}$	$5.18\times 10^{6}$
CPCNN5	96.67	32*64*128*196*896	1×11,7,5,3	$2.55\times 10^{5}$	$1.68\times 10^{7}$

## Data Availability

The training data supporting the conclusions of this article are not available for reasons of subjects’ personal privacy and patents. We will protect the privacy and patent rights of subjects to the fullest extent possible. The model of the subjects was public.

## Abbreviations

The following abbreviations are used in this manuscript:

AUC	Area Under Curve
CC	Chest compression
CCD	CC depth
CPR	Cardiopulmonary resuscitation
CNN	Convolutional neural network
1D-CNN	One-dimensional CNN
DNN	Deep neural networks
DAE	Autoencoder
DAENet	DAE network
ECS	Emergency care simulator
FLOPs	Floating point operations
IR-UWB	Ultrasonic/Impulse radio-ultra wideband
KNN	K-Nearest Neighbor
LSTM	Long short-term memory
ROC	Receiver operating characteristic curve
